# Single nuclear RNA sequencing of terminal ileum in patients with cirrhosis demonstrates multi-faceted alterations in the intestinal barrier

**DOI:** 10.1186/s13578-024-01209-5

**Published:** 2024-02-19

**Authors:** Xixian Jiang, Ying Xu, Andrew Fagan, Bhaumik Patel, Huiping Zhou, Jasmohan S. Bajaj

**Affiliations:** 1 Division of Gastroenterology, Hepatology and Nutrition, Virginia Commonwealth University and Richmond VA Medical Center, 1201 Broad Rock Blvd., Richmond, VA USA; 2 Department of Microbiology and Immunology, Virginia Commonwealth University and Richmond VA Medical Center, 1220 East Broad Street, Richmond, VA 23298 USA

**Keywords:** Hepatic encephalopathy, Infections, Decompensation, Mucus layer, Rifaximin, Defensin

## Abstract

Patients with cirrhosis have intestinal barrier dysfunction but the role of the individual cell types in human small intestine is unclear. We performed single-nuclear RNA sequencing (snRNAseq) in the pinch biopsies of terminal ileum of four age-matched men [56 years, healthy control, compensated, early (ascites and lactulose use) and advanced decompensated cirrhosis (ascites and rifaximin use)]. Cell type proportions, differential gene expressions, cell-type specific pathway analysis using IPA, and cellular crosstalk dynamics were compared. Stem cells, enterocytes and Paneth cells were lowest in advanced decompensation. Immune cells like naive CD4 + T cells were lowest while ITGAE + cells were highest in advanced decompensation patients. *MECOM* had lowest expression in stem cells in advanced decompensation. Defensin and mucin sulfation gene (PAPSS2) which can stabilize the mucus barrier expression were lowest while IL1, IL6 and TNF-related genes were significantly upregulated in the enterocytes, goblet, and Paneth cells in decompensated subjects. IPA analysis showed higher inflammatory pathways in enterocytes, stem, goblet, and Paneth cells in decompensated patients. Cellular crosstalk analysis showed that desmosome, protease-activated receptors, and cadherin-catenin complex interactions were most perturbed in decompensated patients. In summary, the snRNAseq of the human terminal ileum in 4 subjects (1 control and three cirrhosis) identified multidimensional alteration in the intestinal barrier with lower stem cells and altered gene expression focused on inflammation, mucin sulfation and cell–cell interactions with cirrhosis decompensation.

**Supplementary Information:**

The online version contains supplementary material available at 10.1186/s13578-024-01209-5.

## Introduction

Alterations in the gut-liver axis are associated with the progression of chronic liver disease and cirrhosis [1, 2]. Studies have focused on defining the role of the microbiome, local and systemic immune response, as well as intestinal and mucus barrier in cirrhosis progression [1, 3, 4]. These changes have been associated with the development and progression of complications such as hepatic encephalopathy (HE), and spontaneous bacterial peritonitis (SBP) [5–7]. While there have been recent single-cell studies focusing on the liver in early-stage cirrhosis and compared to colon in healthy individuals, the impact of later stages of cirrhosis on the intestinal mucosa needs to be explored in humans [8, 9]. This is particularly relevant in the small intestine, where there is a high potential for bacterial translocation and worsening of systemic inflammation [1]. Moreover, in cirrhosis, there is a decrease in the intestinal mucus layer, which allows greater access to the epithelium by pathogenic bacteria [10, 11]. The main component of mucus, mucin, is produced by goblet cells [12]. Mucin is a large glycoprotein and undergoes stabilization with sulfation [13]. Sulfation of biomolecules in the gut enhances the barrier function by increasing the resistance to bacterial and other enzymatic degradation of mucus and detoxifies potentially harmful substances such as secondary bile acids [14]. There is emerging evidence that the use of medications such as rifaximin could prevent mucin degradation by suppressing bacterial sialidase [15]. Moreover, successful fecal microbiota transplant in patients with cirrhosis significantly increased the proportion of sulfated bile acids [16]. However, evaluation of the interplay of intestinal genes involved in mucus production, stabilization, and defensin production, as well as sulfation across the spectrum of human cirrhosis, is needed.

We hypothesized that individuals with decompensated cirrhosis exhibit a distinctive snRNAseq signature within the small intestinal mucosa. This signature is particularly centered around sulfation and mucin production in goblet cells and Paneth cells, as well as heightened inflammatory expression. This distinct profile is expected to differentiate these patients from both healthy controls and individuals with compensated cirrhosis.

## Materials and methods

After IRB approval, we performed prepped colonoscopies for research in age and sex-matched healthy controls, patients with compensated and decompensated cirrhosis. Healthy controls were free of chronic diseases and were not on any prescription medications. Cirrhosis was diagnosed using liver biopsy or signs of decompensation, or evidence of varices on endoscopy or radiological examination in patients with chronic liver disease. Decompensation was defined as prior or current ascites; early decompensation was defined as ascites with lactulose, which means one HE episode, while advanced decompensation was ascites with rifaximin use, which signifies multiple HE episodes. None of the subjects had any polyps, signs of inflammatory bowel disease or any other colonic or terminal ileal pathology detected during this examination. Pinch biopsies of the terminal ileum were flash-frozen until single-nuclei analysis was performed. We initially started with 4 in each category but as seen below (results), only one sample in each category was successful due to high (> 10%) mtDNA contamination in the rest. Single nucleus RNA sequencing and analysis were conducted by Singulomics Corporation (https://singulomics.com/, Bronx NY). In summary, frozen human colon biopsy tissue samples were homogenized and lysed with Tween-20 in RNase-free water for nuclei isolation. The isolated nuclei were purified, centrifuged, and resuspended in PBS with BSA and RNase Inhibitor. The nuclei isolated were loaded to 10 × Genomics Chromium Controller to encapsulate single nuclei into droplet emulsions following the manufacturer’s recommendations (10 × Genomics, Pleasanton, CA). Library preparation was performed according to the instructions in the Chromium Next GEM 3’ Single Cell Reagent kits v3.1. Amplified cDNAs and the libraries were measured by Qubit dsDNA HS assay (Thermo Fisher Scientific, Wilmington, DE) and quality assessed by TapeStation (Agilent Technologies, Santa Clara, CA). Libraries were sequenced on a NovaSeq instrument (Illumina, San Diego, CA), and reads were subsequently processed using 10 × Genomics Cell Ranger analytical pipeline (v6.1.2) and the human reference genome GRCh38 with introns included in the analysis. Dataset aggregation was performed using the cellranger aggr function normalizing for the total number of confidently mapped reads across libraries. The target was to capture 5000 nuclei per sample with a sequencing depth of 200 million read pairs per sample (e.g., 40,000 read pairs per cell).

### Statistics

The snRNA-Seq results for the human intestinal samples were stored in separate feature-barcode matrices for each respective sample, with each element in the matrices storing the expression level of one specific RNA in one sequenced nucleus. Seurat 4.0(44), a powerful R package designed for the exploration of scRNA-seq and snRNA-seq data was employed to analyze the feature-barcode matrices. The matrices were converted and combined into a Seurat object with a complete barcode, experimental group, and gene expression data. After the expression levels were then preprocessed with log normalization, the gene expression profile of each nucleus underwent a clustering process based on principal component analysis (PCA). PCA focused on the important genes that differentiate the expression profiles of all nuclei the most while filtering out the rest so the expression profiles consisting of thousands of genes can be compared efficiently. Based on the result of PCA, k-nearest neighbors (k-NN) were calculated and clusters were determined through shared nearest neighbor (SNN). The clusters were then visualized in a 2D plot with Uniform Manifold Approximation and Projection (UMAP) technique. All the cells within the same cluster have similar expression profiles and thus usually belong to the same cell type. Critical gene markers, such as RBP2, ANPEP, FABP2 for enterocytes, STAB2 for endothelial cells, and DEFA5 for Paneth cells, etc. were used to identify the cell types and visualized using dot plots. Clusters with high mitochondrial RNA contents were identified to be contaminated and subsequently removed from the nuclei pool for reclustering until the remaining nuclei were relatively clean. With cell types identified, the cell type population sizes were then extracted to calculate the cell type percentage compositions of each sample. Differential gene expressions (DGEs) were also conducted for specific cell types. The DGE lists were then used for cell-type specific pathway analysis in IPA QIAGEN. To gain further insight into the cellular crosstalk dynamics of each sample, CellChat package in R was also employed. CellChat package can be used to infer cell–cell interaction based on ligand-receptor interactions, predict major signaling inputs and outputs for cells and how those cells and signals coordinate for functions using network analysis and pattern recognition approaches [45]. It also offers options for the visualization of cellular crosstalk patterns (Additional file 1).

### Study approval

All study activities were approved by the Richmond VA Institutional Review Board and all subjects provided written informed consent before study activities were initiated.

## Results

### Subjects

We performed snRNA-seq using the pinch biopsies of the terminal ileum from 12 men. However, biopsies from only 4 men had mtDNA < 10% in their sample results (Additional file 2: Fig S3). These were age-matched (56 years); the highest MELD score was in the advanced decompensated patient [14] vs. early decompensation [9] vs. compensated cirrhosis [6]. Both decompensated patients had ascites controlled with diuretics with early one being on lactulose and the advanced one also being on rifaximin.

### SnRNASeq cell types

**Fig. 1:**
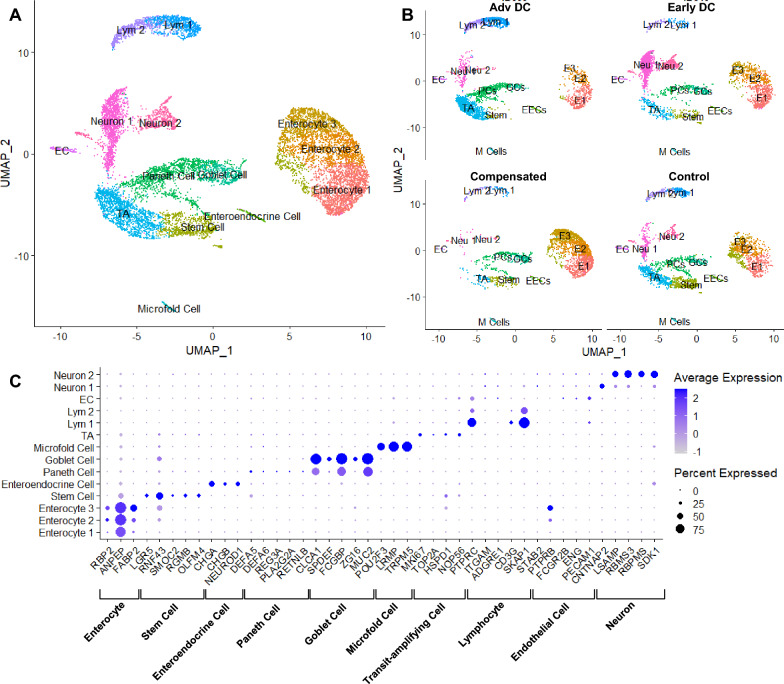
(**A**) Uniform Manifold Approximation and Projection (UMAP) Visualization of the Clustering of All 11350 Nuclei sequenced from All Patient Samples, (**B**) UMAP Visualization of Cell Population from Individual Samples and (**C**) Dotplot for Cell Type Identification Markers Each dot in **A** & **B** represents one nucleus, with its position differentiated by the expression levels of the principal components. 14 clusters were isolated in total and were identified to be the major intestinal cell types based on markers shown in **C**. There was no emergence of novel cell subtypes based on diseased conditions and the differences between the cell populations of all the samples can be characterized by the change of population compositions. For example, the advanced decompensated patient had more lymphocytes, transit-amplifying cells and less enterocytes compared to other samples with less severe conditions

SnRNAseq successfully identified ten major cell types within the intestine (Fig. 1A, B). The estimated number of cells from Cell Ranger are 7180, 3792, 4333, and 4424 nuclei, respectively. The estimated mean reads per cell are 24,597, 43,634, 54,407, and 55,770 read pairs, respectively. Using cell-type specific markers (Fig. 1C), we identified major changes in individual cell types in different disease stages (Table 1). Notably, a substantial reduction in stem cell population was evident in all 3 cirrhosis patients in comparison to the control subject. The percentages of Paneth cells, lymphocytes and TA were much higher, but the percentage of goblet cells was lower in advanced decompensated patients compared to the remaining groups. The percentage of neurons in the early decompensated-associated biopsy was significantly higher than in the rest groups.

**Table 1:** Major changes in individual cell types in different disease stages.

	Advanced decompensation		Early decompensation		Compensated cirrhosis		Control	
	Count	Percent (%)	Count	Percent (%)	Count	Percent ()%)	Count	Percent (%)
Endothelial cell	61	2.01	27	0.88	5	0.20	34	1.25
Enterocyte 1	339	11.18	473	15.44	579	22.77	294	10.84
Enterocyte 2	123	4.06	245	8.00	464	18.25	306	11.29
Enterocyte 3	5	0.16	156	5.09	679	26.70	78	2.88
Enteroendocrine cell	47	1.55	20	0.65	26	1.02	32	1.18
Goblet cell	53	1.75	111	3.62	174	6.84	97	3.58
Lym 1	422	13.92	71	2.32	38	1.49	186	6.86
Lym 2	326	10.75	58	1.89	58	2.28	127	4.68
Microfold cell	27	0.89	14	0.46	28	1.10	29	1.07
Neuron	151	4.98	1070	34.92	20	0.79	183	6.75
Neuron 2	26	0.86	329	10.74	51	2.01	141	5.20
Paneth cell	557	18.37	148	4.83	214	8.42	215	7.93
Stem cell	47	1.55	155	5.06	146	5.74	584	21.54
TA	848	27.97	187	6.10	61	2.40	405	14.94
Total	3032	100.00	3064	100.00	2543	100.00	2711	100.00tata

#### Subgroups

We identified three subgroups of enterocytes. There is a significant loss of enterocyte 2 in the advanced decompensation group (4.06%) compared to controls (11.29%). The marker genes used to identify these cells are also listed in (Additional file 1: Table S1).

Furthermore, we successfully identified lymphocyte subgroups, specifically Lym1 and Lym2, with Lym1 further divided into four subgroups (Additional file 2: Fig S1). Among these, Tc1 (26.30%) and CD8 + Effector memory T Cells (CD8 + TEM) (62.56%) exhibited significant increases in the advanced decompensation group compared to controls (Table 2). Notably, there was a marked reduction in Naive CD4 + T cells (1.9%) within the advanced decompensation group in contrast to controls (96.24%) (Table 2). Lym2 was also segmented into three subgroups. In the advanced decompensation group, there was a notable elevation in ITGAE + cells (90.49%) compared to controls (5.51%) (Table 2), while Central Memory CD4 + T (CD4 + Tcm) cells displayed a decrease (Additional file 2: Fig S2).

**Table 2:** Further separation of immune cells

	Advanced decompensation		Early decompensation		Compensated cirrhosis		Control	
	Count	Percent (%)	Count	Percent (%)	Count	Percent (%)	Count	Percent (%)
Lym1								
CD8^+^TEM	264	62.56	24	33.80	29	76.32	6	3.23
Naive CD4^+^T	8	1.90	31	43.66	3	7.89	179	96.24
Tc1	111	26.30	1	1.41	0	0	0	0
NKT	39	9.24	15	21.13	6	15.79	1	0.54
Lym2								
ITGAE^+^	295	90.49	35	60.34	9	15.52	7	5.51
Central Memory CD4^+^T	11	3.37	16	27.59	1	1.72	115	90.55
CD8^+^ Anergic T	20	6.13	7	12.07	48	82.76	5	3.94

##### Inflammatory genes

**Fig. 2:**
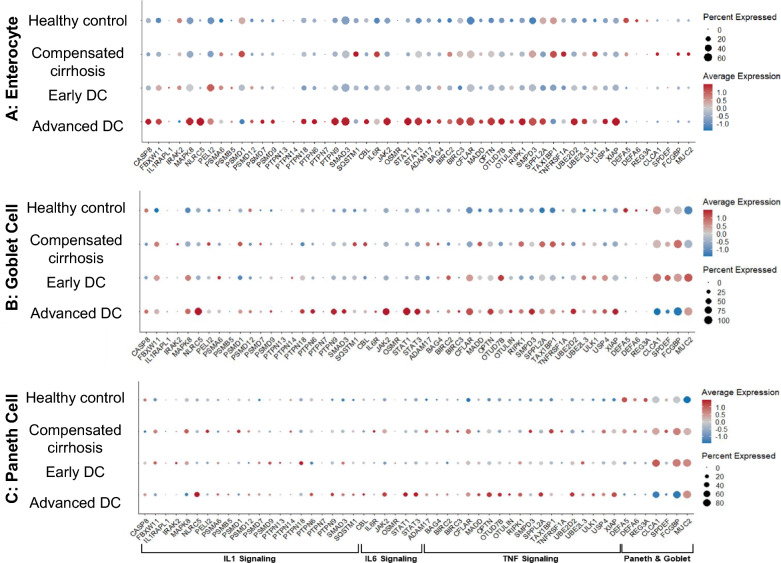
Dotplots of Genes from Major Pro-Inflammatory Pathways and Paneth and Goblet Cell Markers in (**A**) Enterocytes, (**B**) Goblet Cells and (**C**) Paneth Cells. The major inflammatory pathways selected to be visualized were IL1 signalling, IL6 signalling, and TNF signalling, while the visualization of Paneth and Goblet cell markers reflected their production levels of important mucus components like mucin and defensin. There were heightened expression levels of pro-inflammatory genes across all three cell types in the advanced decompensated patient. Compensated and early decompensated patients had similar expression levels of the pro-inflammatory genes while the healthy control had the lowest. The healthy control also had the highest expressions for genes associated with defensin production, which gradually decreased for other patients with worsening cirrhosis. On the other hand, the genes associated with the production of mucin were least expressed in the healthy control, they increased with worsening cirrhosis

We analyzed the major genes related to inflammatory pathways and mucus production in enterocytes, goblet cells and Paneth cells. As shown in Fig. 2, the IL1, IL6 and TNF-related genes were significantly upregulated in the enterocytes, goblet cells and Paneth cells across all decompensated subjects, especially those with advanced decompensation compared to healthy control. The healthy control also had the highest expressions for genes associated with defensin production, which gradually decreased for other patients with worsening cirrhosis.

##### Sulfation genes

**Fig. 3:**
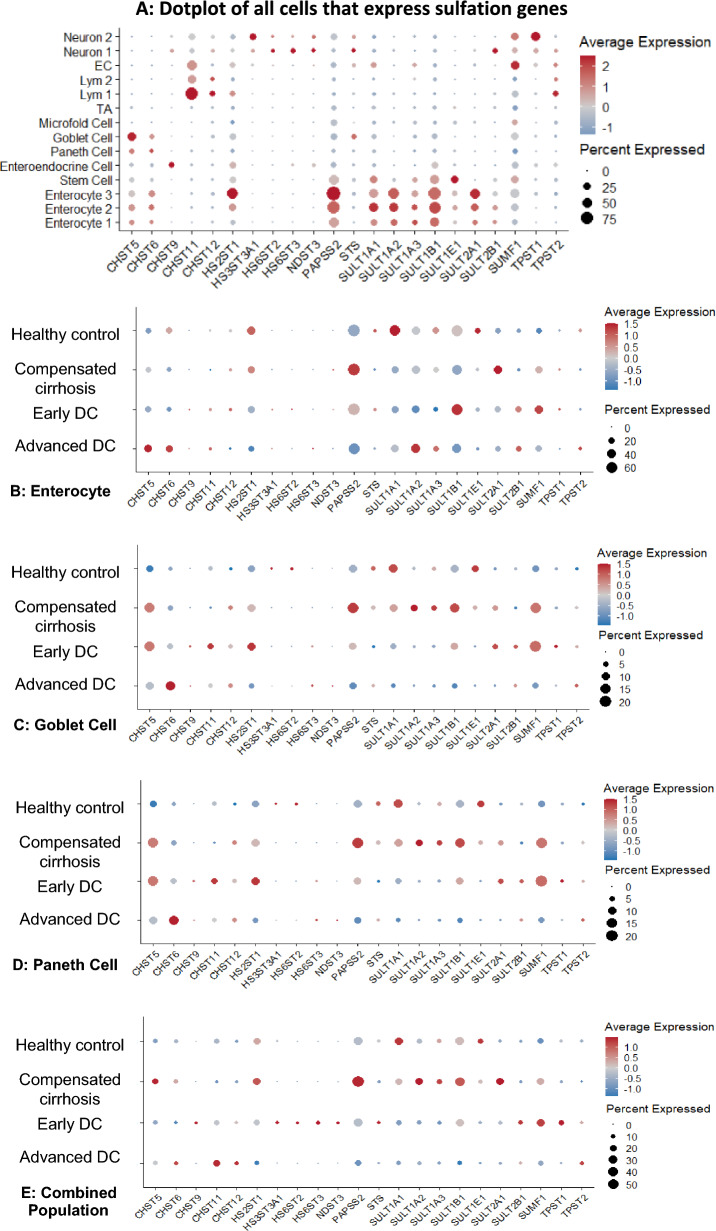
Dotplots of Major Sulfation Related Genes in (**A**) All Cell Population Divided by Cell Types, (**B**) Enterocytes, (**C**) Goblet Cells, (**D**) Paneth Cells, and (**E**) All Cell Population Divided by Samples. Enterocytes were shown to be the main cell type expressing sulfation related genes especially those from the sulfotransferase (SULT) family (**A**). As shown in **B**–**E**, the compensated sample expresses the most sulfation related genes, demonstrating a major increase from the healthy control, while the expression levels of these genes decreased in decompensated patients as the degree of decompensation progressed

Most sulfation genes were found in the enterocytes, followed by some in the lymphocytes and Paneth cells (Fig. 3). The major sulfation gene, PAPSS2 (3’-phosphoadenisine 5’-phosphosulfate synthase 2) had the greatest expression in enterocytes, along with sulfotransferase 1A2-3 (SULT1A2-3). Heparan sulfation genes were relatively sparse but mostly found in enterocytes. Chondroitin sulfation genes, which are important for mucus stabilization, were mostly expressed in goblet cells, lymphocytes, and some enterocyte lineages. When individual subjects and cells were considered, PAPSS2 expression in enterocytes and goblet cells was lower in the advanced decompensation group versus the other cirrhosis patients. However, cytosolic sulfotransferases SULTA2-3, but not SULTB1, expression was higher in the enterocytes of the decompensation group. Intriguingly, with respect to glycosaminoglycan sulfation, Heparan sulfate sulfotransferase (HS2ST1) expression was lower, while chondroitin sulfate sulfation genes (CHST5-6) were higher in all cells in the advanced decompensation group versus the others.

##### Paneth cells

The Paneth cell markers that were most expressed across cells were defensin alpha 5 (DEFA5), DEFA6 and regenerating family member3 (REG3A), which were spread across several cell types as expected. The strongest expression was in Paneth cells for DEFA6, while most stem cells expressed this gene. DEFA6 and REG3A were comparatively not as strongly expressed. The greatest expression of defensin-coding genes was in controls, compensated cirrhosis versus decompensated patients (Figs. 2 and 5).

##### Goblet cells

Regarding goblet cell markers, calcium-activated chloride channel regulator 1 (CLCA1), mucin 2 (MUC2) and fibroblast growth factor receptor substrate 2 binding protein (FGCBP) showed the highest expression and prevalence in the goblet and Paneth cells overall. Zymogen granule protein 16 (ZG16) and SAM pointed domain ETS factor (SPDEF) had comparatively lower strength and prevalence of expression. It's notable that within the advanced decompensation group, there was a discernible decrease in the expression of goblet cell markers FGCBP, CLCA1, and SPDEF, which play roles in goblet cell differentiation, promoting mucus regeneration, and dampening inflammation. However, in contrast, the expression of MUC2, which is involved in mucin production, was elevated in both decompensated groups.

##### Stem cells

**Fig. 4:**
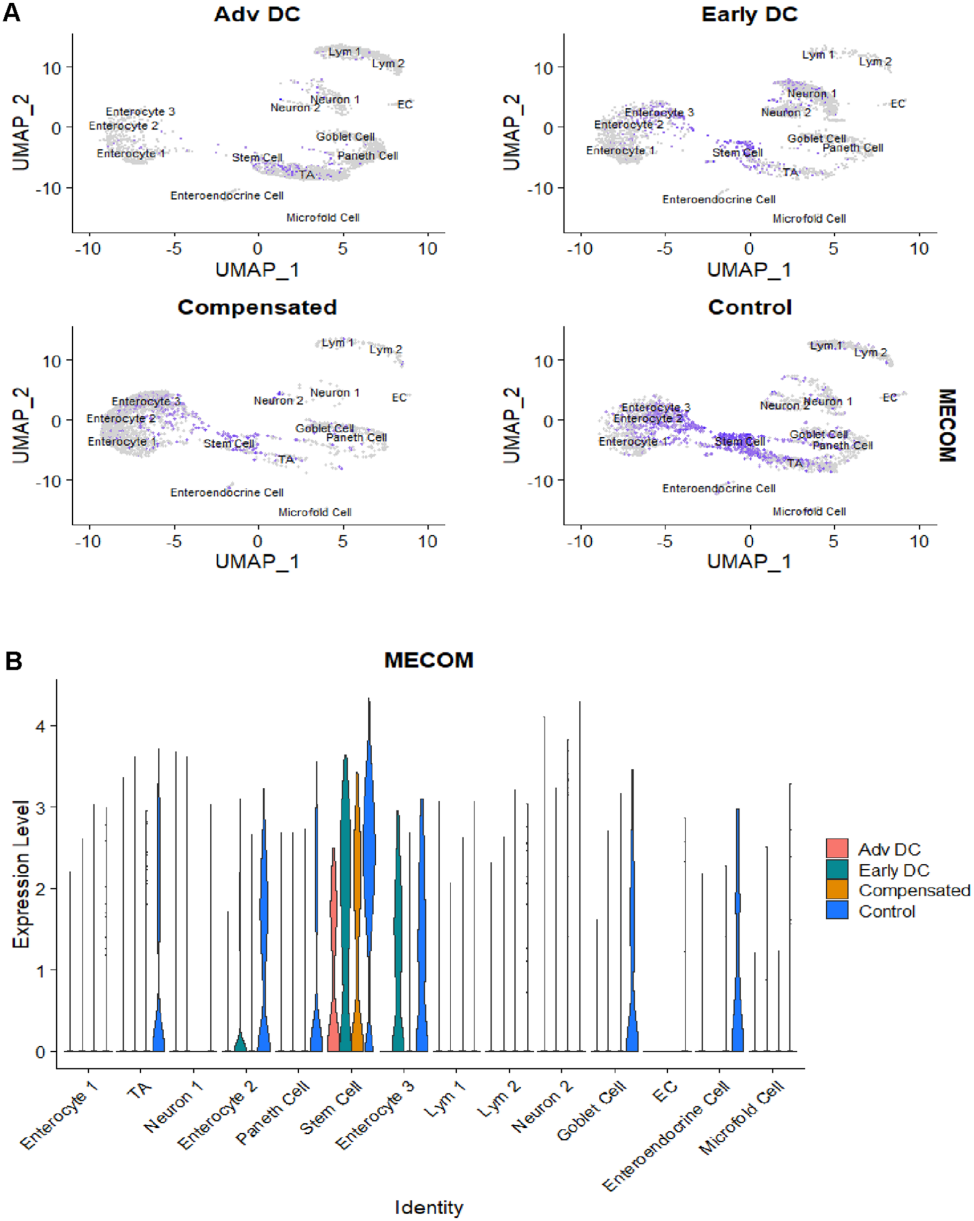
Feature Plot Visualization (**A**) and VlnPlot Visualization (**B**) of MECOM expression in 4 groups. MECOM was mainly expressed in stem cell and was less in advanced decompensated patient than control group

There was a significant loss of stem cells in all patients with cirrhosis compared to controls. To figure out the differences of stem cells in4 groups, we compared the expression of cell markers between advanced decompensation group and control group. *MECOM* was significantly downregulated in cirrhosis groups and the expression is lowest in advanced decompensation group while highest in control group (Fig. 4).

#### IPA analysis

**Fig. 5:**
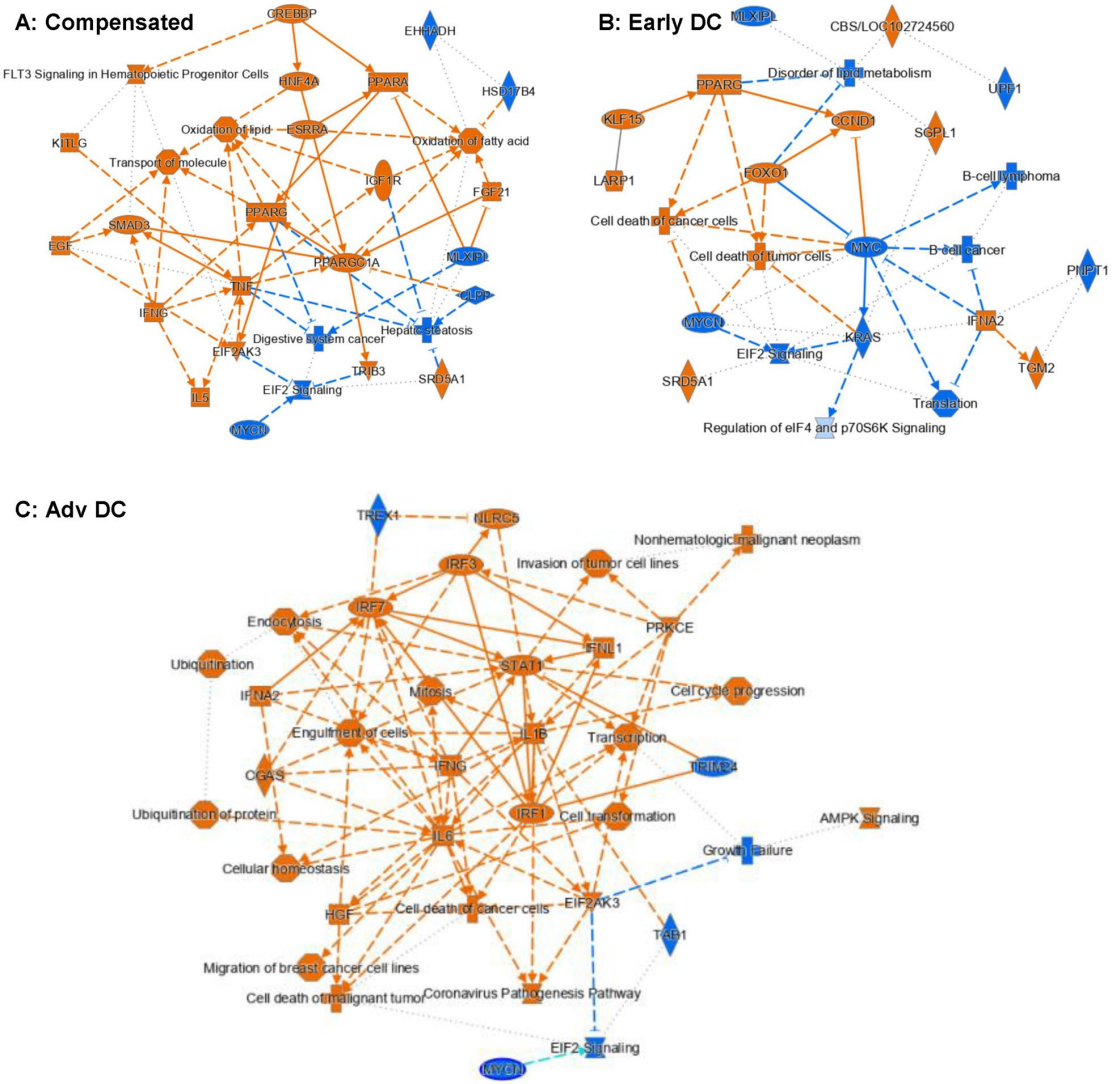
IPA Analysis Results for Enterocyte-Specific Differential Gene Expressions for (**A**) Compensated vs Healthy Control, (**B**) Early Decompensated vs Healthy Control and (**C**) Advanced Decompensated vs Healthy Control. The pathway maps show the potential interactions between major transcription factors, pathways, and biological functions that were differentially expressed or predicted to be regulated. Orange represents upregulation and blue represents downregulation. Numerous regulated pathways can be observed in these pathway maps. A notable pattern is the upregulation of inflammation and immune faction related pathways in all three samples as compared the healthy control, including TNF and IFNG in A, IFNA2 in B, and IFNG, IL6, IL1B in C

**Enterocytes (**Fig. 5**): **IPA analysis showed significant activation of inflammatory pathways (TNF, IFNG), PPARG/A with gut-liver signaling pathways (FGF21, IGF1R, CREBP) and fatty acid and lipid oxidation in compensated cirrhosis versus controls (Fig. 5A). However, the EIF2(eukaryotic initiation factor 2), CLPP (caseinolytic mitochondrial matrix peptidase proteolytic subunit), and MLXIPL (max-like protein X interacting protein-like) were significantly downregulated in the compensated patient (Fig. 5A). In early decompensation, again there was higher inflammation (IFNA2) and PPARG with lower MYC and KRAS compared to controls (Fig. 5B). In advanced decompensation, there was the highest activation of inflammatory pathways (IFNG, IL6, IL1B) and interferon regulatory genes with lower MYC compared to controls (Fig. 5C).

**Fig. 6:**
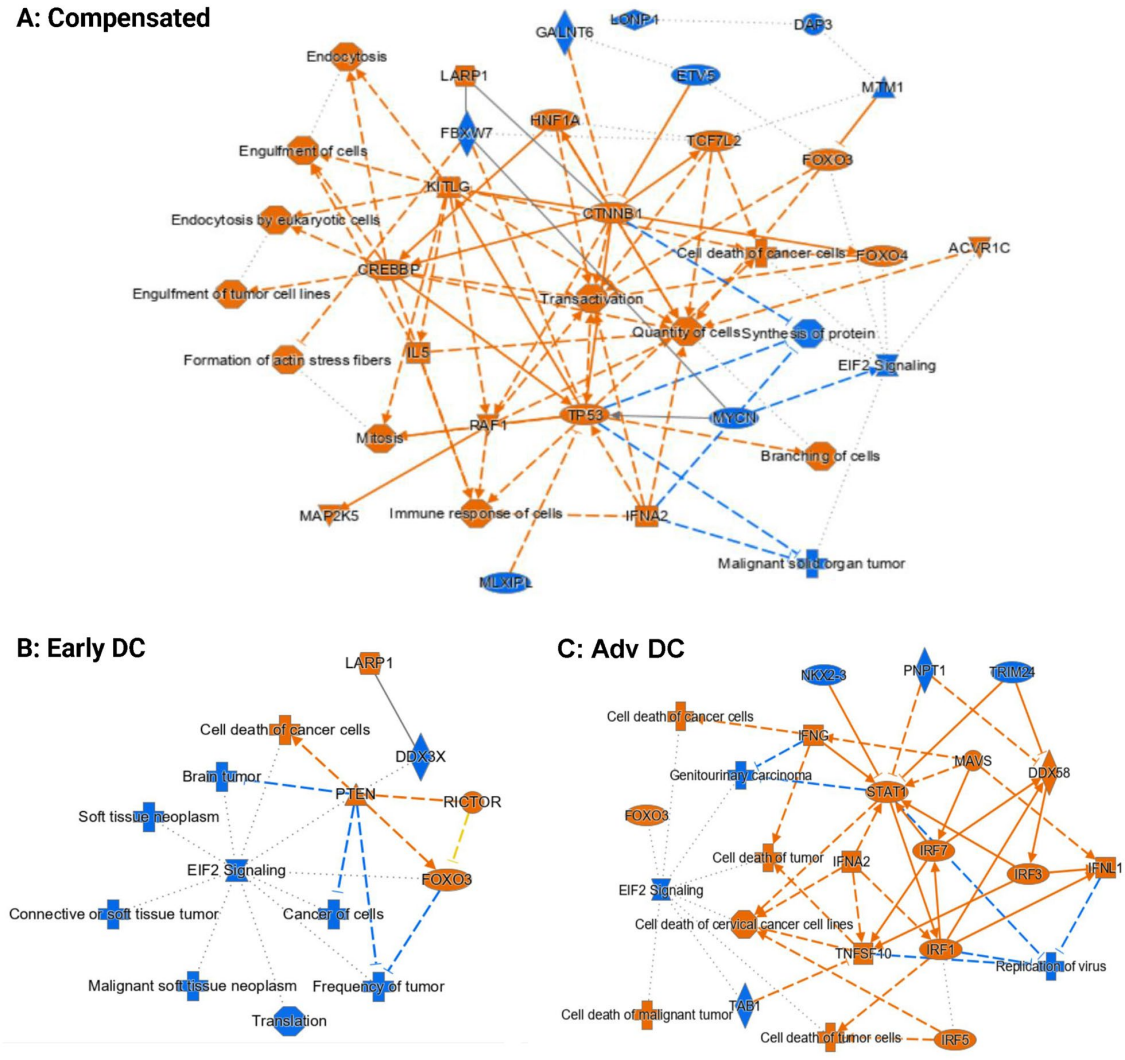
IPA Analysis Results for Goblet Cell-Specific Differential Gene Expressions for (**A**) Compensated vs Healthy Control, (**B**) Early Decompensated vs Healthy Control and (**C**) Advanced Decompensated vs Healthy Control. The pathway maps show the potential interactions between major transcription factors, pathways, and biological functions that were differentially expressed or predicted to be regulated. Orange represents upregulation and blue represents downregulation. The goblet cells from all cirrhosis patients notably exhibited lower EIF2 signalling levels and higher expressions of the transcription factor FOXO3. Higher expressions of multiple IRFs (interferon regulatory factor) were detected in the advanced decompensated patient (**C**)

**Goblet cells (**Fig. 6**): **Advanced decompensated patients had higher STAT1, IFNA2, IFNG and IRF (interferon regulatory factor) expression compared to controls. All cirrhosis patients had lower EIF2 signaling levels and higher expressions of the transcription factor FOXO3 compared to controls. Compensated group also showed activation of endocytosis, engulfment, and immune response versus controls. Both RICTOR (a rapamycin-insensitive component of mTOR) and LARP1 (La ribonucleoprotein domain family member 1) were activated in the early decompensated patient.

**Fig. 7:**
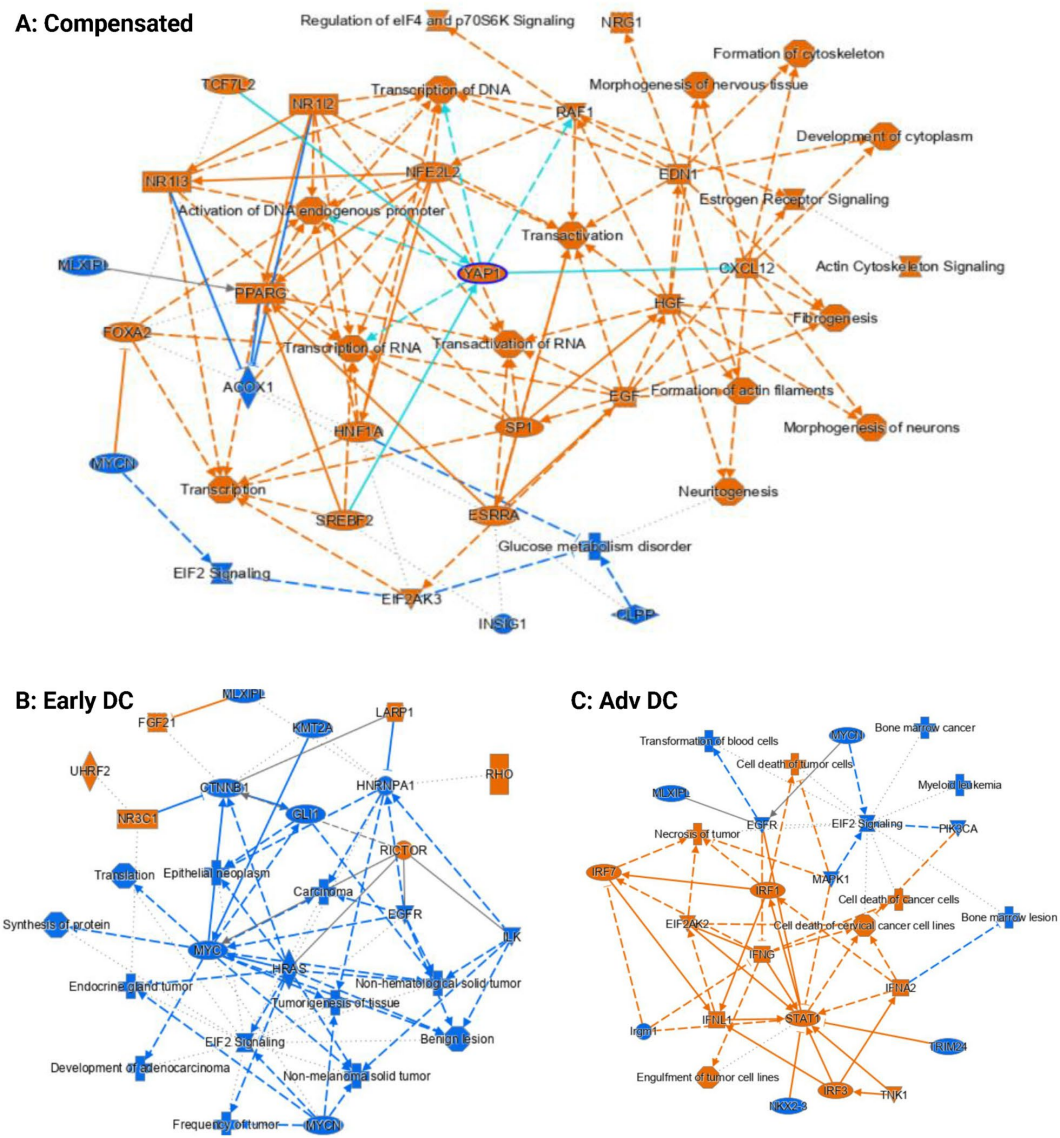
IPA Analysis Results for Paneth Cell-Specific Differential Gene Expressions for (**A**) Compensated vs Healthy Control, (**B**) Early Decompensated vs Healthy Control and (**C**) Advanced Decompensated vs Healthy Control. The pathway maps show the potential interactions between major transcription factors, pathways, and biological functions that were differentially expressed or predicted to be regulated. Orange represents upregulation and blue represents downregulation. Upregulations of EGF (epidermal growth factor) and HGF (hepatocyte growth factor) can be observed in the Paneth cells from the compensated patients, leading to the upregulations of multiple pathways related to cell growth (**A**). The trend was reversed in both decompensated patients with the downregulation of EGFR (epidermal growth factor receptor) and cancer-related transcription factors MYC or MYCN (**B**, **C**). However, just like in goblet cells, the downregulation of EIF2 signalling can be observed in the Paneth cells from all three cirrhosis patients

**Paneth cells (**Fig. 7**): **Similar to goblet cells, in advanced decompensated patients, the signaling pathways of STAT1, IFNA2, IFNG and IRF were activated compared to controls. Upregulations of EGF (epidermal growth factor) and HGF (hepatocyte growth factor) can be observed in the Paneth cells from the compensated patients, leading to the upregulations of multiple pathways related to cell growth (Fig. 7A).The trend was reversed in both decompensated patients with the downregulation of EGFR (epidermal growth factor receptor) and cancer-related transcription factors MYC or MYCN (Fig. 7B, C). Early decompensated group again had activated RICTOR and LARP1 signaling pathways. PPARG and cell and neuronal synthesis pathways were also activated in compensated patients versus controls. In addition, EIF2 signaling pathway was downregulated in all cirrhosis patients compared to control similar to what was observed in goblet cells.

**Fig. 8:**
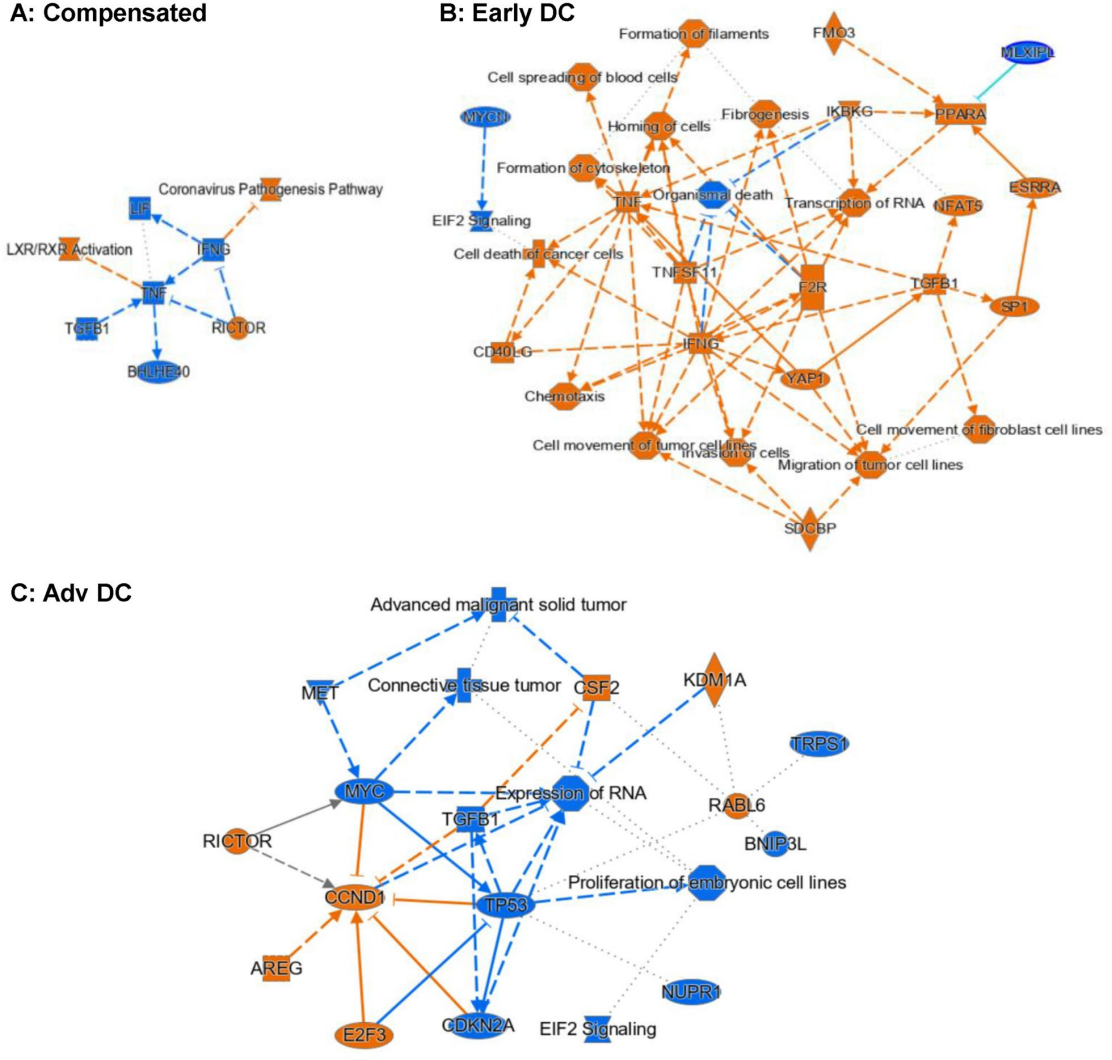
IPA Analysis Results for Stem Cell-Specific Differential Gene Expressions for (**A**) Compensated vs Healthy Control, (**B**) Early Decompensated vs Healthy Control and (**C**) Advanced Decompensated vs Healthy Control. The pathway maps show the potential interactions between major transcription factors, pathways, and biological functions that were differentially expressed or predicted to be regulated. Orange represents upregulation and blue represents downregulation. Not many significant changes were noticed in the compensated sample besides the downregulation of two inflammation related genes TNF and IFNG (**A**). Both genes were however upregulated in the early decompensated patient along with growth promoting pathways related to YAP1 and TGFB1 (**B**). In the advanced decompensated patient, TGFB1 was downregulated (**C**)

**Stem cells (**Fig. 8**): **Compared to controls, LXR/RXR and RICTOR signaling pathways were activated in compensated cirrhosis but inflammatory pathways (IFNG, TNF) were downregulated. This pattern changed with decompensation; the inflammatory pathways (TNF, IFNG) and PPARA, along with cell movement, structure, and growth promoting transcription coregulator YAP1were activated. In advanced decompensated patients, the signaling pathways related to RICTOR, CCND1, E2F3, AREG, CSF2, KDM1A and RABL6 were activated, but the signaling pathways of TGFB1, MYC, MET, CDKN2 were inhibited [17].

#### Cell chat analysis

**Fig. 9:**
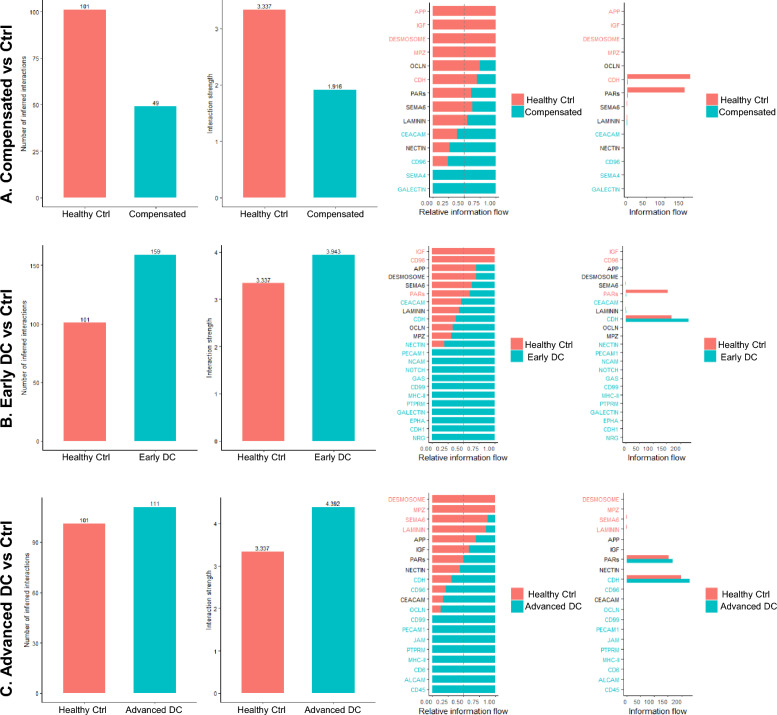
Comparisons of Numbers of Inferred Interactions, Total Interaction Strength, and Information Flow of Significant Pathways for (**A**) Healthy Control vs Compensated, (**B**) Healthy Control vs Early Decompensated, and (**C**) Healthy Control vs Advanced Decompensated. The numbers of inferred interactions are total numbers of cellular interactions between all cell types detected by CellChat while the interaction strength is a relative value generated by CellChat to compare the interaction intensity between samples. The compensated sample had a lower interaction level than the healthy control as shown in **A** while both decompensated samples had higher interaction levels as shown in **B** & **C**

**Fig. 10:**
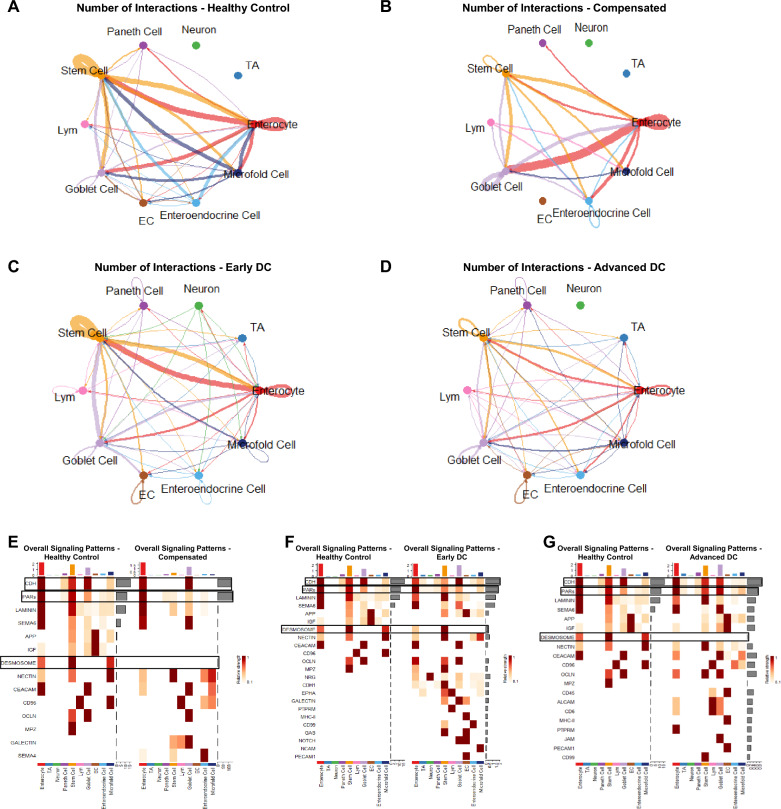
Circle Plot for Inferred Cellular Interaction Dynamics in (**A**) Healthy Control, (**B**) Compensated, (**C**) Early Decompensated, (**D**) Advanced Decompensated Samples and Side-by-side Comparison Heatmaps for the Overall Signalling Strength of All Cell Types in Major Identified Signalling Pathways for (**E**) Compensated, (**F**) Early Decompensated, and (**F**) Advanced Decompensated Samples vs Healthy Control. The circle plots offer detailed looks into the numbers of interactions happening between each cell type. The color of each curve corresponds to its outgoing cell type while the size of each curve reflects the relative number of interactions. Notable changes observed include a decrease of interactions between enterocytes and stem cells can be observed in the compensated sample as compared to the healthy control (**A**, **B**). Interactions involved enterocytes further decreased in both decompensated samples but interactions involving Paneth cells and transit-amplifying cells increased. The side-by-side heatmaps visualize the relative strength of each cell type’s participation in major detected signalling pathways. The loss of CDH and PARs signalling in stem cells in compensated and early decompensated patients can be observed when compared to the healthy control. However, the expressions of both signalling pathways returned to stronger levels in the advanced decompensated patient. The loss of DESMOSOME signalling can be observed in all diseased samples compared to the healthy control, mainly in enterocytes, stem cells, and microfold cells

To further understand the cell–cell communications across different disease conditions, we performed CellChat analysis. As shown in Fig. 9A, the compensated cirrhosis exhibited decreased inferred interactions and interaction strength compared to healthy control. However, both early and advanced decompensated patients displayed increased interred interactions and interaction strength compared to the control (Fig. 9B, C). The cell type-specific interactions were also significantly changed in compensated and decompensated patients compared to the control. As shown in Fig.10A, B, the signals from enterocytes to goblet cells were increased, but the signals from macrophages to goblet cells, stem cells, and enterocytes were significantly reduced in both compensated and decompensated patients. The heatmaps of overall signaling patterns also showed that the desmosome signaling pathways were significantly reduced in enterocytes, stem cells and microfold cells in compensated and advanced decompensated patients but increased in early decompensated patients (Fig. 10E–G). Both CDH (Cadherin-catenin complex) and PARs (protease-activated receptor) pathways were suppressed in compensated cirrhosis but upregulated in decompensated patients compared to healthy controls. In controls, stem cells showed higher CDH signaling compared to compensated patients, while similar cell types were involved in early compensation and a higher proportion of goblet cells were involved in CDH in advanced decompensation. PAR signaling was higher in the compensated group in the stem cells and goblet cells, while the reverse pattern for goblet cells was seen in advanced decompensation. Similar to CDH, early decompensated group did not show differences in PAR signaling versus controls.

## Discussion

We successfully identified different types of cells in the terminal ileum of patients with cirrhosis using snRNAseq, albeit in a small number of subjects. As disease progresses in cirrhosis, there are significant shifts in the different cell populations, gene expression profiles, and signaling pathways. These major changes identified include mucus production and stabilization with sulfation, as well as inflammatory and intestinal barrier integrity. These findings underscore the intricate landscape of cellular changes that coincide with cirrhosis progression; however, these were only performed in tissues from 4 subjects.

Impairment of the intestinal barrier, which is characterized by structural and functional changes leading to bacterial translocation, is a central event in the progression of cirrhosis [1, 18]. The complex alterations underlying this impairment involve a multifaceted interplay encompassing various factors. These encompass gut luminal factors, including microbiome, medication effects and exogenous insults such as high-fat diet and alcohol consumption). Additionally, mucosal alterations, including changes in mucus layer thickness, adherent bacteria, and immune activation), play a significant role. Furthermore, this intricate process involves neuro-hormonal and immune aspects, including both local GI and systemic immune response [7]. Cirrhosis, especially during the phase of decompensation, exerts a comprehensive impact on all these aspects of the intestinal barrier, including increased permeability, altered gut microbiome, intestinal inflammation, and changes in mucus production [18]. The current study results underline the multi-dimensional changes in the intestinal barrier, including mucus layer, stem cells, and sulfation, which worsen as cirrhosis progresses.

However, most human studies have either primarily evaluated stool, luminal contents or duodenal or colonic samples [19–22]. In contrast, our study uniquely focuses on the terminal ileum (TI), which is one of the main sites for several host-microbial interactions as well as immune-microbial interfaces [23, 24]. The results show a reduction in stem cells in the TI in cirrhosis versus controls, which worsened across decompensated patients. To gain a more comprehensive understanding of the observed reduction in stem cells, we conducted a detailed analysis and compared the gene expression differences in stem cells between advanced decompensation group and control group. One gene, *MECOM,* was the topmost gene and exhibited the most substantial downregulation within the advanced decompensation group compared to the control group. *MECOM* (MDS1 and EVI1 complex locus) plays an important role in the endothelial cell differentiation and angiogenesis through self-renewal and differentiation of stem cells, by acting as a transcriptional regulator and by influencing epigenetic modifications [25]. Moreover, this is related to the *VEGF* signaling pathway and is associated with ischemia reperfusion. This highlighted the potential significance of reduced MECOM expression within the advanced decompensation group. Such a reduction could potentially compromise the cellular processes responsible for maintaining mucosal oxygenation and balance of ischemia, thereby potentially exacerbating endotoxemia and further impairing the integrity of the intestinal barrier [25].

We further delved into the immune cell clusters by dissecting the Lym clusters to gain a deeper understanding. Intriguingly, the advanced decompensation group showed lower naive CD4^+^T cells and higher proportion of ITGAE^+^ cells. This observation aligns with a prior study wherein lower naive CD4^+^Tcells were observed in the peripheral blood of patients with advanced liver fibrosis [26]. Our findings extend this observation into the ileal mucosa of patients with cirrhosis. While the mechanism is unclear, this could be due to an altered gut-liver axis and increased immuno-senescence [26]. Additionally, we found a higher proportion of ITGAE^+^cells (also known as CD103), that were CD8 positive in the advanced decompensation group. CD103 mediates tissue retention of ^+^CD8^+^T cells through E-cadherin and show a greater expression in the liver of patients with autoimmune hepatitis and in the intestine with TCR triggering and Th17-related gene expression in Crohn’s disease [27–29]. The observed changes in immune cell proportions can be influenced by several factors in cirrhosis. These could be related cirrhosis-associated chronic hepatic and systemic inflammation leading to the activation and differentiation of T cells, resulting in a decrease naïve CD4 + T cells. Activated T cells, especially effector memory and central memory CD4 + T cells may increase in response to chronic inflammation. Immune exhaustion due to constant immune activation and exposure to antigens characterized by increased expression of inhibitor receptors on T cells could be contributory and result in upregulation of ITGAE (integrin alph E, also known as CD103) in exhausted T cells. While not studied here, the gut microbial changes in cirrhosis could also play a role in this altered immune activation and cirrhosis-related dysregulation of immune checkpoints and immune regulatory mechanisms [30]. Immune checkpoints, such as programmed cell death protein 1 (PD-1) and cytotoxic T lymphocyte-associated protein 4 (CTLA-4) could affect this immune profile. Finally, portal hypertension can influence immune cell trafficking and localization within the gut associated lymphoid tissue, which can affect the proportions of various immune cell subsets [30]. To explore the inflammatory genes further, in concordance with prior studies, there was a significant upregulation of inflammatory genes (IL1, IL6 and TNF) in patients with cirrhosis [31]. These were related to enterocytes and not stem cells, goblet, or Paneth cells. Prior studies of scRNA seq have shown lower stem cell fractions in the liver in patients with cirrhosis, however, our findings of a similar pattern in the terminal ileum are novel [8, 9]. This is striking because unlike inflammation, which was more significant in decompensated patients, relatively low proportion of stem cells were found even in the compensated patient. This indicates an effect of cirrhosis that affects the intestine even before decompensation sets in and could have implications for the health of the intestinal barrier through impaired regeneration.

Another important layer of the intestinal barrier that is varyingly affected in chronic liver disease is the mucus layer [18]. A recent study in human tissues showed that in cirrhosis, especially those with spontaneous bacterial peritonitis (SBP), there was a reduction in the mucus layer. However, this study did not examine sulfation or genes involved in mucus secretion [10]. A lower intestinal mucus thickness could potentially increase the contact with intraluminal and mucosal bacteria, which have been shown to be enriched in pathobionts in patients with cirrhosis. Another study of pre-cirrhotic individuals who were drinking alcohol showed a thicker mucus layer in the upper intestine, likely as a compensatory mechanism [32]. Our results showed a higher MUC2 expression in goblet cells and Paneth cells in cirrhosis compared to controls, like the alcohol-related mouse study mentioned above, which could be a compensatory mechanism. This was accompanied by lower FcGBP and SPDEF expression in advanced decompensated TI Paneth and goblet cells [33, 34]. Both FcGBP and SPDEF are involved in the differentiation of goblet and Paneth cells. This suggests a complex interpla governing the regulatory processes of goblet and Paneth cell differentiation. Mucus sulfation, which stabilizes mucus against degradation, is more complex [35, 36]. The major donor of sulfur in the intestine is PAPSS2, which is the major rate-limiting step for this high-affinity, low-capacity system of sulfation [37]. Interestingly, PAPSS2 exhibited the greatest expression in enterocytes overall. However, when individual subjects were considered, PAPSS2 expression in enterocytes and goblet cells was lower in the advanced decompensation TI versus the others. While the mechanism is unclear, evidence suggests that inflammation in the liver suppresses PAPSS2 expression and intestine specific PAPSS2 knockout mice are more prone to intestinal inflammation [38, 39]. Our findings extend these insights to the terminal ileum of humans with cirrhosis. This suppression of PAPSS2 could reduce the substrate for subsequent reactions involving sulfation of several macromolecules, including lipids, proteins, and glycosaminoglycan (GAGs) such as chondroitin and heparan as well as xenobiotics and drugs. GAGs have multiple beneficial properties in normal intestinal homeostasis [40]. Heparan sulfation genes were relatively sparse but mostly found in enterocytes and HS2ST1 gene expression was lower in advanced patients versus the rest in all cells. Heparan sulfate has several roles in the small intestinal barrier, including binding cells to extracellular matrix and modulating the response to microbiota [41, 42]. Chondroitin sulfation genes were mostly expressed in goblet cells, lymphocytes and some enterocyte lineages and were higher in decompensated subjects, again likely a compensatory mechanism. Finally, SULT2A1, which are predominantly involved in cholesterol and bile acid sulfation in humans, were mostly expressed in enterocytes and was lower in decompensated TI cells [37, 43]. This has implications for the intestinal barrier since sulfated bile acids are less likely to be toxic to ileal membrane integrity. Lower sulfated bile acids have been found in advanced decompensated patients, which are restored by liver transplant and by fecal microbial transplant [16, 44]. These multiple changes in sulfation need to be interpreted in the context of the lower sulfur donor availability as the disease progresses through lower PAPSS2 expression in enterocytes, in validation experiments with animal models and larger groups of patients.

The IPA analysis provided further insights into the inflammatory landscape among cirrhosis patients, highlighting the substantial activation of inflammatory pathways, including activation of lymphocytes, mononuclear leukocytes, and natural killer cells, lymphocyte migration, and interferon regulatory factors (IRFs). In advanced compensated patients, there is significant activation of IFNr and IRFs (IRF1, IRF3 and IRF7)a in enterocytes. IRFs can be activated by various signals and play a critical role in regulating the expression of genes involved in the immune response [45]. Across all cirrhosis patients, a noteworthy downregulation of the EIF2 signaling pathway in enterocytes, goblet cells, Paneth cells and stem cells emerged. EIF2 plays an essential role in controlling protein synthesis and maintaining cellular homeostasis [46, 47]. Downregulation of EIF2 has been implicated in intestinal inflammation and cell death [46, 47]. This may contribute to the loss of stem cells in advanced decompensated patients. Overall, these findings indicate the intricate interplay of inflammatory pathways and cellular processes, potentially influencing the dynamics of stem cells and disease progression in cirrhosis patients.

CellChat analysis identified compelling shifts in cell–cell communication. Apart from compensated group, where the number of inferred interaction pathways was lower than controls, the other cirrhosis groups display higher interaction pathways compared to the control. The desmosome-related pathway had higher information flow in control sample versus all cirrhosis groups, especially in enterocytes and stem cells. This is likely reflective of the stronger intestinal barrier that is promoted by desmosome activation in healthy people compared to cirrhosis and could be potentially related to the lower stem cell population in the diseased group. On the other hand, two other pathways, CDH and PARs in stem cells, were nuanced, with the higher flow in controls vs. compensated and early decompensated, but the expressions of both signaling pathways returned to stronger levels although they were still lower than controls in the advanced decompensated patient. PARs in the intestinal stem cells are promoters of cell regeneration and affect cell proliferation and migration from the crypts [48]. CDH-related genes are associated with cell–cell adhesion through E-cadherin and β-catenin interactions, which is critical for intestinal barrier integrity. In intestinal stem cells, E-cadherin could help maintain contact between cells at the bottom of the crypts and reduces towards the top to enhance migration [49]. Desmosomes are key cell–cell junction components which connect adjacent epithelial cells through intermediate filaments. Alteration of desmosome function and cadherin-catenin complexes can result in gaps or discontinuities between epithelial cells, compromising barrier function. PARs are a family of cell-surface receptors that respond to proteolytic enzymes, such as thrombin and trypsin. Catherin-Catenin complex is involved in cell adhesion and plays a crucial role in maintaining the integrity of the intestinal epithelial barrier. The relatively higher expression in the advanced decompensated patient could be due to a compensatory mechanism or could be an intestinal barrier improvement due to the rifaximin this patient was taking. Regardless, the differential expression of enterocyte and stem cell desmosome, and stem cells CDH and PARs, which affect migration and differentiation, show that the intestinal barrier changes across cirrhosis are multidimensional. In cirrhosis, liver dysfunction contributes to systemic inflammation. The disrupted intestinal barrier function due to desmosome and cadherin-catenin perturbations may exacerbate systemic inflammation, potentially worsening cirrhosis-related complications. Additionally, a dysregulated PAR interactions may lead to excessive inflammation and tissue damage in the intestines.

Our study interpretation is limited by one sample analyzed per group, which was in part due to mitochondrial contamination in the remaining samples. However, these samples are relatively inaccessible given the location and the challenges in performing colonoscopies in advanced decompensated patients with cirrhosis. We used snRNA sequencing rather than the scRNA sequencing, which has some key differences. Given the limitations of snRNAseq could not delineate immune cells other than lymphocytes. We are not able to delineate the impact of superadded medications vis-à-vis the cirrhosis itself since this is a cross-sectional study and it would be unethical to withdraw these patients [50]. Prior studies with lactulose and rifaximin have mixed results on microbiome in pre/post studies in compensated cirrhosis, where these medications are not indicated [51, 52]. Further clinical trials are needed with these medications to determine if these changes in snRNAseq expression because of medications versus disease progression. Also, we did not perform individual validation experiments, animal model experiments, or evaluate the gut microbiome. However, several of these factors have been studied in other parts of the intestine in animal models of cirrhosis but our experience translates these into the human distal small intestinal level.

In conclusion, our study, using snRNA sequencing of the terminal ileum in patients with cirrhosis, has unveiled substantial shifts in gene expression within key components of the intestinal barrier, including enterocytes, goblet cells and Paneth cells compared to control. This multidimensional change spans various critical aspects, including cell–cell adherence, mucus secretion and sulfation, bile acid sulfation, as well as stem cell markers. Collectively, these findings contribute to an enhanced understanding of the intricate interplay within the context of the alternated gut-liver axis and its role in driving cirrhosis progression. The insights garnered from this study pave the way for a more comprehensive investigation of the pathophysiology underlying cirrhosis, holding potential implications for the development of targeted interventions in the future.

### Supplementary Information

**Additional file 1: Table S1.** Intestinal cell markers

**Additional file 2: Figure S1.** (A)Dotplot for Cell Type Identification Markers in different clusters.(B)Feature Plot Visualization of Cell Type Identification Markers(C) UMAP Visualization of the Clustering of subgroups of Lym1 from All Patient Samples and (D)UMAP Visualization of Cell Population from Individual Samples. Lym1 was separated into 4 subgroups, and based on the markers which are showed in A & B, The subgroups were identified. There was a significant loss in naive CD4+T in advanced decompensated patient. **Figure S2.** (A)Dotplot for Cell Type Identification Markers in different clusters in Lym2.(B)Feature Plot Visualization of Cell Type Identification Markers (C) UMAP Visualization of the Clustering of subgroups of Lym2 from All Patient Samples and (D) UMAP Visualization of Cell Population from Individual Samples. Lym2 was separated into 3 subgroups, and based on the markers which are showed in A & B, The subgroups were identified. ITGAE was the top gene in the cluster and this cluster was identified as ITGAE+ cells. **Figure S3.** Violin plot depicting the distribution of mitochondrial DNA levels across most nuclei after filtering

## Data Availability

Due to IRB restrictions, data are not available.
