# Supplementary material for: Single nuclear RNA sequencing of terminal ileum in patients with cirrhosis demonstrates multi-faceted alterations in the intestinal barrier
Source: Cell Biosci. 2024 Feb 19;14:25. doi: 10.1186/s13578-024-01209-5 (PMC10875857; doi:10.1186/s13578-024-01209-5)
Supplement: Supplementary file 1 — Additional file 1: Table S1. Intestinal cell markers [file 13578_2024_1209_MOESM1_ESM.docx]

**Supplementary data**

1. Supplementary Table
2. Supplementary Figures 1 and 2 legends

**Table S1: Intestinal cell markers**

| Endothelial Cell | STAB2 ,PTPRB ,FCGR2B ,ENG ,PECAM1 |
| --- | --- |
| Enterocyte | RBP2 ,ANPEP ,FABP2 |
| BEST 4 Enterocyte | BEST4 ,OTOP2 ,CA7 |
| Colonocyte | CA2 ,SLC26A2 ,FABP1 |
| Enteroendocrine Cell | CHGA ,CHGB ,NEUROD1 |
| Goblet Cell | CLCA1 ,SPDEF ,FCGBP ,ZG16 ,MUC2 |
| Microfold Cell | POU2F3 ,LRMP ,TRPM5 |
| Paneth Cell | DEFA5 ,DEFA6 ,REG3A ,PLA2G2A ,RETNLB |
| Stem Cell | LGR5 ,RNF43 ,SMOC2 ,RGMB ,OLFM4 |
| Transit-amplifying (TA) | MKI67 ,TOP2A ,HSPD1 ,NOP56 |
| Sulfation genes | CHST5, CHST6, CHST9, CHST11, CHST12, HS2ST1, HS3ST3A1, HS6ST2, HS6ST3, NDST3, PAPSS2, STS, SULT1A1, SULT1A2, SULT1A3, SULT1B1, SULT1E1, SULT2A1, SULT2B1, SUMF1, TPST1, TPST2 |
| Inflammatory genes |  |
| TNF | ADAM17, BAG4, BIRC2, BIRC3, CASP8, CFLAR, MADD, OPTN, OTUD7B, OTULIN, RIPK1, SMPD3, SPPL2A, TAX1BP1, TNFRSF1A, UBE2D2, UBE2L3, ULK1, USP4, XIAP |
| IL-6 | CBL, IL6R, JAK2, OSMR, STAT1, STAT3 |
| IL-1 | CASP8, FBXW11, IL1RAPL1, IRAK2, MAPK8, NLRC5, PELI2, PSMA6, PSMB5, PSMD1, PSMD12, PSMD7, PSMD9, PTPN13, PTPN14, PTPN18, PTPN6, PTPN7, PTPN9, SMAD3, SQSTM1 |

**Figure S1 Legend**

**(A)Dotplot for Cell Type Identification Markers in different clusters.(B)Feature Plot Visualization of Cell Type Identification Markers(C) UMAP Visualization of the Clustering of subgroups of Lym1 from All Patient Samples and (D)UMAP Visualization of Cell Population from Individual Samples.** Lym1 was separated into 4 subgroups, and based on the markers which are showed in **A** & **B**, The subgroups were identified. There was a significant loss in naive CD4+T in advanced decompensated patient.

**Figure S2 Legend**

**(A)Dotplot for Cell Type Identification Markers in different clusters in Lym2.(B)Feature Plot Visualization of Cell Type Identification Markers (C) UMAP Visualization of the Clustering of subgroups of Lym2 from All Patient Samples and (D) UMAP Visualization of Cell Population from Individual Samples.** Lym2 was separated into 3 subgroups, and based on the markers which are showed in **A** & **B**, The subgroups were identified. ITGAE was the top gene in the cluster and this cluster was identified as ITGAE+ cells.

**Figure S3 Legend**

Violin plot depicting the distribution of mitochondrial DNA levels across most nuclei after filtering
