# Supplementary material for: Single nuclear RNA sequencing of terminal ileum in patients with cirrhosis demonstrates multi-faceted alterations in the intestinal barrier
Source: Cell Biosci. 2024 Feb 19;14:25. doi: 10.1186/s13578-024-01209-5 (PMC10875857; doi:10.1186/s13578-024-01209-5)
Supplement: Supplementary file 2 — Additional file 2: Figure S1. (A)Dotplot for Cell Type Identification Markers in different clusters.(B)Feature Plot Visualization of Cell Type Identification Markers(C) UMAP Visualization of the Clustering of subgroups of Lym1 from All Patient Samples and (D)UMAP Visualization of Cell Population from Individual Samples. Lym1 was separated into 4 subgroups, and based on the markers which are showed in A & B, The subgroups were identified. There was a significant loss in naive CD4+T in advanced decompensated patient. Figure S2. (A)Dotplot for Cell Type Identification Markers in different clusters in Lym2.(B)Feature Plot Visualization of Cell Type Identification Markers (C) UMAP Visualization of the Clustering of subgroups of Lym2 from All Patient Samples and (D) UMAP Visualization of Cell Population from Individual Samples. Lym2 was separated into 3 subgroups, and based on the markers which are showed in A & B, The subgroups were identified. ITGAE was the top gene in the cluster and this cluster was identified as ITGAE+ cells. Figure S3. Violin plot depicting the distribution of mitochondrial DNA levels across most nuclei after filtering [file 13578_2024_1209_MOESM2_ESM.pptx]

## Slide 1
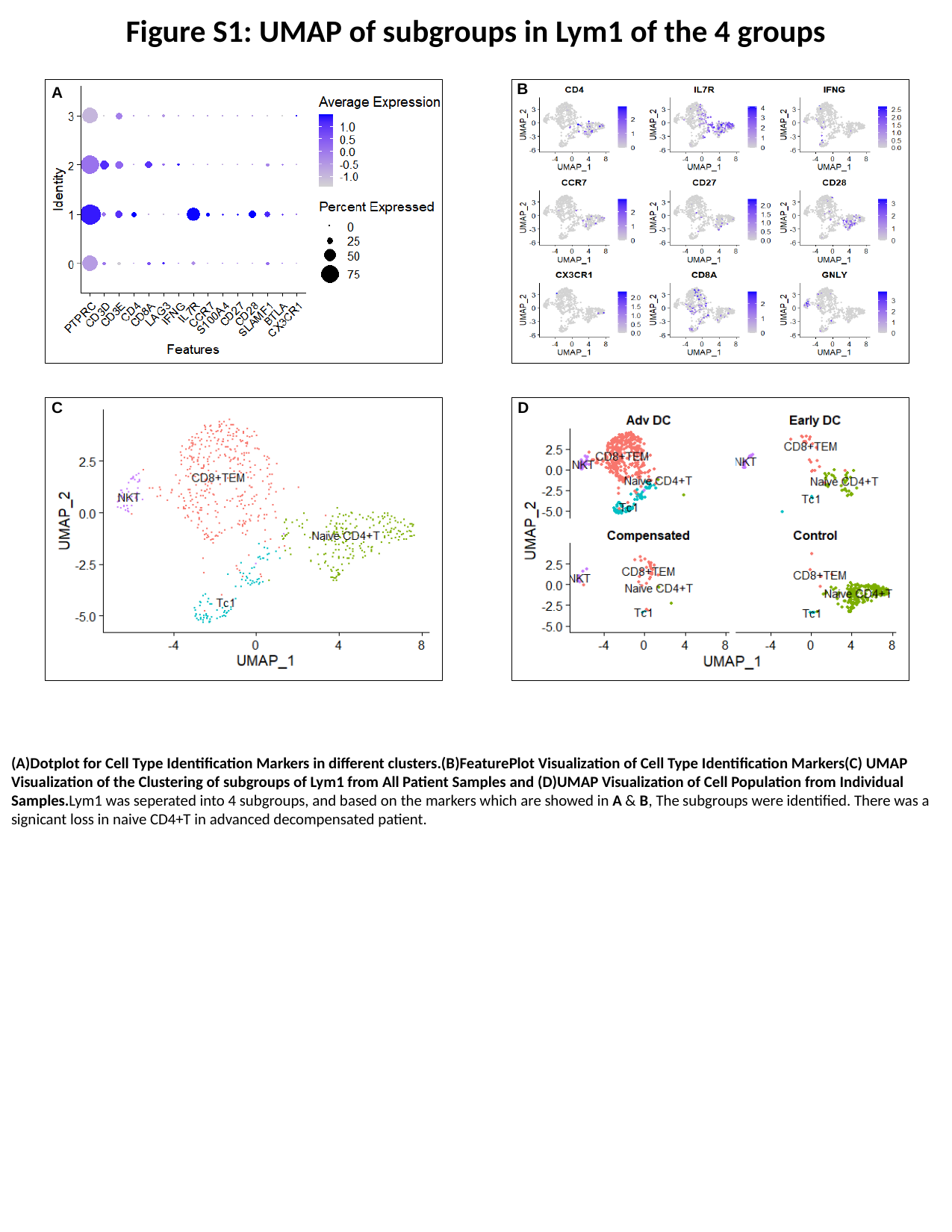

Figure S1: UMAP of subgroups in Lym1 of the 4 groups
B
A
C
D
(A)Dotplot for Cell Type Identification Markers in different clusters.(B)FeaturePlot Visualization of Cell Type Identification Markers(C) UMAP Visualization of the Clustering of subgroups of Lym1 from All Patient Samples and (D)UMAP Visualization of Cell Population from Individual Samples.Lym1 was seperated into 4 subgroups, and based on the markers which are showed in A & B, The subgroups were identified. There was a signicant loss in naive CD4+T in advanced decompensated patient.

## Slide 2
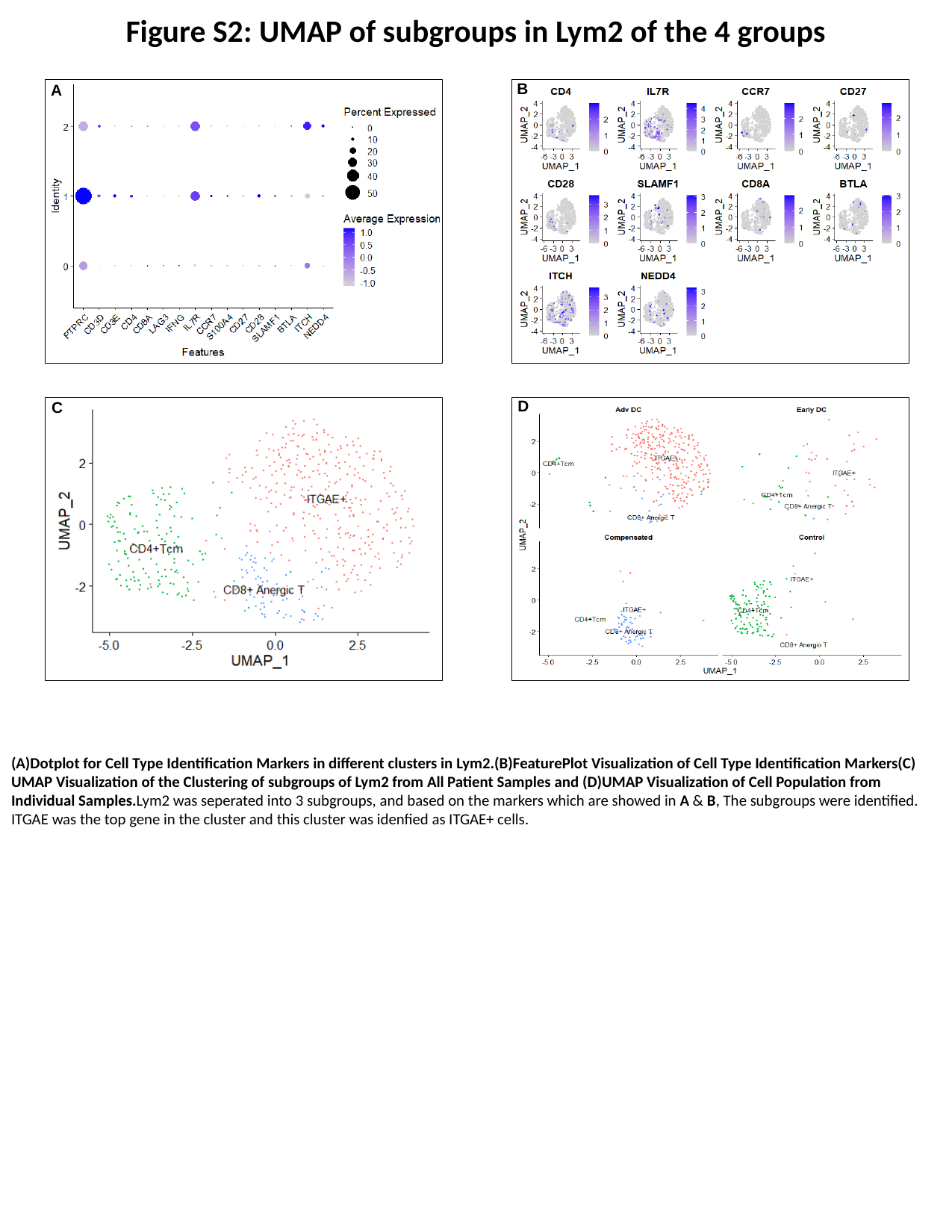

Figure S2: UMAP of subgroups in Lym2 of the 4 groups
B
A
D
C
(A)Dotplot for Cell Type Identification Markers in different clusters in Lym2.(B)FeaturePlot Visualization of Cell Type Identification Markers(C) UMAP Visualization of the Clustering of subgroups of Lym2 from All Patient Samples and (D)UMAP Visualization of Cell Population from Individual Samples.Lym2 was seperated into 3 subgroups, and based on the markers which are showed in A & B, The subgroups were identified. ITGAE was the top gene in the cluster and this cluster was idenfied as ITGAE+ cells.

## Slide 3
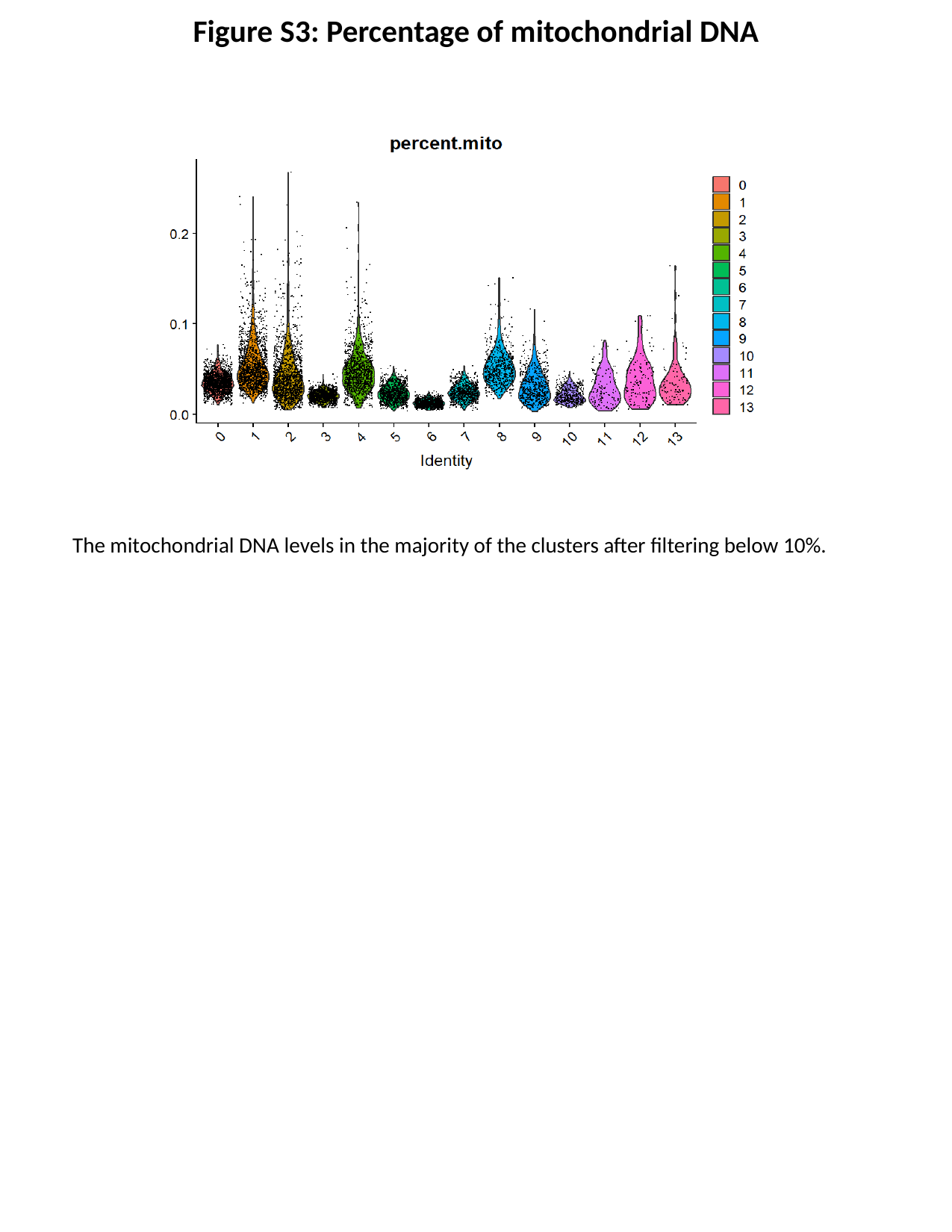

Figure S3: Percentage of mitochondrial DNA
The mitochondrial DNA levels in the majority of the clusters after filtering below 10%.
